# Detailed clinical characteristics of musical hallucinations in 81 patients

**DOI:** 10.1007/s00415-026-13958-z

**Published:** 2026-07-06

**Authors:** Daphne L. Kerklaan, Jan Dirk Blom, Ouarda Bouachmir, Jan Adriaan F. Coebergh

**Affiliations:** 1 Parnassia Psychiatric Institute, The Hague, the Netherlands; 2 Faculty of Social and Behavioural Sciences, Leiden University, Leiden, the Netherlands; 3 Department of Psychiatry, University Medical Center Groningen, Groningen, the Netherlands; 4 Department of Neurology, Ashford/St. Peter’s Hospital, Chertsey, UK; 5 Department of Neurology, St. George’s Hospital, Blackshaw Road, London, UK

**Keywords:** Deafferentiation, Epilepsy, Hearing loss, Multimodal hallucination, Perceptual release, Spatial localisation

## Abstract

**Introduction:**

Musical hallucinations are perceptions of music without an external source. Approximately 500 publications on this topic have appeared over the past 35 years. Prior literature has largely consisted of case reports and small series, with only limited systematic studies on the characterisation of mixed pathology, relation to hearing loss, spatial localisation, and multimodal features.

**Materials and methods:**

We conducted a retrospective analysis of baseline data from a prospective cohort study of 81 individuals experiencing musical hallucinations. Participants underwent assessment with the Musical Hallucinations (MuHa) Questionnaire—a tailored, non-validated semi-structured survey/interview—alongside additional questionnaires, EEG, neuroimaging, and audiological testing. The present analysis focuses on baseline phenomenological characteristics derived from the MuHa Questionnaire in 80 eligible participants. Analyses were exploratory and descriptive.

**Results:**

Mean age was 65 years, with a slight female predominance. Hallucinations were most often perceived as internal, while external localisation was more common with familiar music and in individuals with (asymmetric) hearing loss. Underlying causes included psychiatric disorders (50%), hearing loss (50%), structural neurological changes (28%), and frequently combinations thereof (28%). A notable new finding was the high prevalence of tinnitus and multimodal hallucinations, involving up to six sensory modalities; these were generally associated with internal localisation, except in isolated hearing loss. Musical content spanned multiple genres, most commonly religious music. Hallucinations were considered burdensome in over 65% of the cases, and most participants reported little control; behavioral strategies such as distraction or listening to external music provided only temporary relief in about 20%.

**Conclusion:**

Musical hallucinations may be more common than previously appreciated within broader multimodal perceptual syndromes and mixed etiologies, suggesting a more distributed pathophysiology than traditionally assumed. Their burden and limited controllability highlight the need for further mechanistic and therapeutic research.

**Supplementary Information:**

The online version contains supplementary material available at 10.1007/s00415-026-13958-z.

## Introduction

*I was hearing the same song over and over again in my head—Zeppelin, of course, I can’t remember which one—and it was driving me* crazy*. Day in, day out, it just wouldn’t stop. I was going around the house with a tape recorder trying to record it and asking [my wife], “Do you hear it? Do you hear it?” I distinctly remember being on the staircase, going down to get a gun to shoot myself—not because I wanted to die, just to make the music stop*.

Alex Van Halen, 2024, p. 188.

A livelier introduction to musical hallucinations than the passage above is hardly imaginable. It stems from *Brothers*, the 2024 bestselling book by the musician, Alex Van Halen, who experienced these phenomena in the context of benzodiazepine withdrawal [1]. This brief characterisation touches upon some of the cardinal aspects of musical hallucinations. They consist of music generated by the brain in the absence of an external source, and they can indeed be highly realistic and burdensome. People may experience them either inside the head (as if through well-balanced ear pods) or outside. [2, 3] As evidenced by Mr. Van Halen, people may have a hard time distinguishing them from actual music. This sets musical hallucinations apart from earworms or “tunes in the head,” i.e. musical phenomena that lack perceptual characteristics and are never mistaken for actual music. Also known as brainworms, intrusive musical imagery, and musical obsessions [3, 4], such tunes in the head are considered very common in the general population [5]. By contrast, musical hallucinations are much rarer than earworms; although not as rare as once thought, with an estimated incidence in elderly populations of around one per 10,000 [6], a reported incidence among people with hearing problems of 5.2% [7], and, in other groups with hearing loss, of 0.86% to 2.5% [8, 9]. Pathophysiologically, these phenomena are attributed to spontaneous activity within the brain network devoted to the perception, storage, and retrieval of music [10], and etiologically to either hypoacusis, brain lesions, epilepsy, psychiatric disorders, or intoxications––or often a combination, which has been less studied [11, 12]. Included in the category of intoxications are also withdrawal syndromes as experienced by Mr. Van Halen [1].

In fact, all the features described above by Mr. Van Halen are characteristic of musical hallucinations. Less characteristic is the patient being a middle-aged rock star quitting a habit of substance use. On the basis of > 500 scientific papers on musical hallucinations published over the past 35 years, typical patients would rather be lonelily and/or isolated elderly women with hearing loss who suddenly start to hear music and realise within 1 or 2 days that it must stem from within their head [10, 13]. In previous reviews of the existing literature on 276 cases, hearing impairment was most commonly present (60.4%) and psychiatric disorders in 39.4%; of note, this was not systematically assessed (Coebergh et al., 2015). The hallucinations themselves are typically described as isolated perceptual phenomena which rarely lead to delusions or other positive symptoms of psychosis [14]. Contentwise, ever since the 2005 publication by Warner and Aziz [6], it is often maintained that they involve predominantly children’s songs, opera, and religious music.

Many people experiencing musical hallucinations are reluctant to consult a health professional, fearing stigmatization, and a diagnosis of psychosis or dementia. However, having difficulty concentrating and sleeping, [8, 15, 16] and suffering from the unrelenting onslaught of music, in the end many become exhausted and depressed [17]. They may also become suicidal or show dangerous behavior, as recounted by Mr. Van Halen, too. As a consequence, they may then present for example to psychiatry, neurology, or otolaryngology. In Mr. Van Halen’s case, the music ceased abruptly within a few days. Perhaps that is the natural course for musical hallucinations in the context of withdrawal syndromes––i.e. that they are fleeting and self-limiting in nature––although not enough data is available to corroborate that. [12, 18–20] The majority of published reports on musical hallucinations involve protracted cases. That may well be the reason why they have a reputation of chronicity and incurability [21], even though successful treatment results after many years have been reported on, too [12, 22, 23].

In the light of his job description as a drummer in a rock and roll band, it is perhaps unsurprising that Mr. Van Halen perceived classic rock rather than the types of music reported on more frequently in scientific literature. That, as well as other discrepancies that we noticed in our clinical practice, made us suspect that the profile outlined above (i.e. of elderly women with hearing loss) may not do justice to the full spectrum of people who experience musical hallucinations. The present paper therefore seeks to describe (a) the phenomenological characteristics of musical hallucinations based on a structured, descriptive dataset, and (b) the people experiencing them. To address these issues, we draw on data from the Dutch *MuHa Study*, to our knowledge the largest prospective study to date on musical hallucinations. In particular whether there are multimodal hallucinations has had limited study [33] and experienced localisation has not had any systematic study. Moreover, there has been no previous study of the relationship between hearing loss and symptoms.

## Materials and methods

The *MuHa Study* was carried out from 2010 through 2023 as a prospective cohort study on musical hallucinations at Parnassia Psychiatric Hospital, in collaboration with the Haga Hospital, both in the Hague (reported on before in Buijk et al.[14]). In 2010, the study received ethical approval from the Ethical Review Board of the Haga Hospital (number: 10–114). Candidates were referred by specialists throughout the Netherlands and screened for eligibility. Inclusion criteria were (1) a history of musical hallucinations, (2) age ≥ 18 years, and (3) proficiency in the Dutch language. During the screening process, special care was taken to include only people who experienced musical hallucination, and to exclude those with earworms and other non-perceptual musical phenomena. After providing informed consent, we took a medical history from those included and administered several questionnaires. A clinical nurse specialist, trainee, or consultant psychiatrist read out the questions in person; when in doubt, either the second or last author were consulted. The instruments used were the *Musical Hallucinations* (*MuHa*) *Questionnaire* (a tailor-made, non-validated questionnaire/semi-structured survey; see Supplement 1) and Dutch translations of the *Launay–Slade Hallucination Scale* (LSHS) [24], the *Schizotypal Personality Questionnaire* (SPQ) [25], the *Hamilton Depression Rating Scale* (HDRS) [26], the *Mini Mental State Examination* (MMSE) [27], the *Childhood Trauma Questionnaire* (CTQ) [28], and the *Life Events Questionnaire* (LEQ) [29]. In addition, we attempted where possible an EEG and a brain MRI (and/or head CT), and carried out audiological testing. EEG was not analysed beyond the recording of presence of interictal epileptiform abnormalities and there was no documentation of last/active presence of musical hallucinations.

After the baseline assessment, participants were invited to take part in annual follow-up sessions. The present study utilised the responses to the *MuHa Questionnaire* at baseline, as well as clinical reports, imaging reports, EEG results, and audiology results.

The data that we extracted from the MuHa database comprised (i) demographic variables (gender, age), (ii) clinical diagnoses, (iii) tone audiometry results, (iv) content of the musical hallucinations (based on the participants’ first assessment, divided into six musical genres), (v) familiarity with the music perceived, (vi) self-reported musicality of the participants, (vii) spatial localisation of the hallucinations (inside or outside the head or different), (viii) co-occurring hallucinations and sensory distortions, and (ix) ensuing burden and self-reported control. We used SPSS 27 for statistical analyses. To assess the presence of statistically significant associations we conducted chi-square tests of independence using a *p*-value of < 0.05. Binary logistic regression models were additionally performed for key outcomes, with age and sex included as covariates. All analyses were exploratory and not pre-specified. Results should therefore be interpreted descriptively rather than confirmatory. Small numbers in some categories make generalizability uncertain.

## Results

### Demographics, symptom onset, and referral

The total number of people that we included was 81; of them, 80 (98.8%) fulfilled our criteria for the present analysis. The participants’ demographics are displayed in Table 1. Their mean age was 65 years (range: 15–95 years, ± 18.9 SD); 58.8% of them were female. On average, females were older, with a mean age of 70 years (± 17.2 SD) versus 58 years (± 18.7 SD) for men (*p* = 0.003). Of the 80 participants, 35 (43.8%) recalled the onset of their musical hallucinations. The mean interval between onset and inclusion to our study was eight years (range: 0–44 years), with an average of 14 years for men and 4 years for women (*p* = 0.11). Most participants were referred from psychiatry (45.0%), followed by neurology (17.5%), general practice (10.0%), and otolaryngology (5.0%). One participant (1.3%) was referred by a geriatrician. In 21.3% of the cases, the referring physician was not recorded.

**Table 1:** Patient demographics and diagnoses

	Male (%)	Female (%)	Total (%)
Number of participants	33 (41.3%)	47 (58.8%)	80 (100.0%)
Mean age (in years)	58	70	65
Psychiatric diagnosis	19 (23.8%)	14 (17.5%)	33 (41.3%)
Hearing loss	6 (7.5%)	11 (13.8%)	17 (21.3%)
Structural neurological	1 (1.3%)	2 (2.5%)	3 (3.8%)
Psychiatric + hearing loss	0 (0.0%)	3 (3.8%)	3 (3.8%)
Structural neurological + hearing loss	4 (5.0%)	13 (16.3%)	17 (21.3%)
Psychiatric + structural neurological + hearing loss	0 (0.0%)	3 (3.8%)	3 (3.8%)
Other diagnosis	1 (1.3%)	0 (0.0%)	1 (1.3%)
No identifiable cause	2 (2.5%)	1 (1.3%)	3 (3.8%)

### Clinical diagnoses

We categorised clinical diagnoses at baseline into three main groups, comprising psychiatric conditions, hearing loss, and structural neurological changes. A dominant psychiatric condition was found in 33 participants (41.3%), solely hearing loss in 17 (21.3%), and solely structural neurological changes in three (3.8%). However, combinations of diagnoses were common too. Seventeen participants (21.3%) were diagnosed with both hearing loss and structural neurological changes, three (3.8%) with both hearing loss and a psychiatric condition, and another three (3.8%) with conditions from all three groups (psychiatric disorder, hearing loss, and structural neurological changes). One participant (1.3%) was diagnosed with neurosyphilis, which did not fall within the predefined categories, and was therefore classified as “other.” In three other cases (3.8%) no identifiable cause could be established. Table 1 shows an overview of these diagnoses, as well as their distribution for males and females. We excluded from the results those participants who were categorised as having an “other diagnosis” or where “no identifiable cause” was found.

Among the participants diagnosed with a psychiatric condition, the most common diagnoses were psychotic disorder (41.0%), depressive disorder (33.3%), and bipolar disorder (5.1%). We reviewed imaging reports and included as structural changes only those with significant atrophy or vascular changes in areas involved in auditory processing, although we realise that this is no definitive evidence of causality. Most common were lacunar infarcts (12 participants, 52.2%) and severe white-matter changes (5 participants, 21.7%), while 1 participant was diagnosed with dementia (4.3%). Finally, three participants (3.8%) had a history of epilepsy, although this was likely causative in only one person, who experienced post-ictal musical hallucinations.

### Audiometry

Tone audiometry results at baseline (8 years after symptom onset, on average) were available for 52 participants (65.0%). Based on the average pure-tone thresholds (PTAs) for the right and left ears, we categorised participants in accordance with the classification of hearing impairment issued by the *World Health Organization* (WHO) (Table 2) [30].

**Table 2:** Hearing impairment, scored according to WHO criteria

Hearing impairment	Males (%)	Females (%)	Total (%)
None (< 20 dB)	8 (10.0%)	6 (7.5%)	14 (17.5%)
Mild (20–34 dB)	5 (6.3%)	1 (1.3%)	6 (7.5%)
Moderate (35–49 dB)	2 (2.5%)	10 (12.5%)	12 (15.0%)
Moderate-severe (50–64 dB)	4 (5.0%)	7 (8.8%)	11 (13.8%)
Severe (65–80 dB)	0 (0.0%)	5 (6.3%)	5 (6.3%)
Profound (> 80 dB)	0 (0.0%)	4 (5.0%)	4 (5.0%)

According to the *American Academy of Otolaryngology-Head and Neck Surgery*, asymmetrical hearing loss is defined as a PTA difference of > 15 dB between ears [31]. Among the 52 participants with available audiometry data, 12 (23.1%) met this criterion, while the remaining 40 (76.9%) did not.

Regarding listening devices, 34 participants (42.5%) were documented hearing-aid users, 37 (46.3%) were not, and on 7 (11.3%) we lacked sufficient data. Among the participants who used hearing aids, 26 (76.5%) had undergone tone audiometry. Of the participants who did not use them, 23 (62.2%) had (Supplementary material, Table S2). However, given the use of hearing aids, it is reasonable to infer that the group without audiometry data had a hearing loss of at least 35 dB in the best ear, which is the minimum hearing loss required for eligibility for a hearing aid in the Netherlands. Based on this assumption, the data were recategorised into two groups: one for hearing loss of < 35 dB (25.0%), and one for hearing loss of ≥ 35 dB (50.0%). When these groups were compared by gender (Supplementary material, Table S2), 13 males and seven females were categorised as having hearing loss of < 35 dB and 32 females and eight males were categorised as having hearing loss of ≥ 35 dB.

### Content

To assess the content of our participants’ musical hallucinations, we made a differentiation based on genre (Table 3). Since almost everyone reported on several genres, the total number of genres scored exceeds the number of participants. Most frequently reported was religious music (18.1%, including Christmas carols, choir music, and gospels), followed by children’s songs and popular songs (each 16.8%). We classified music that did not fit into any of the predefined categories as “other” (10.3%). Some noteworthy examples in the latter group were ad jingles, the Windows start-up sound, and “the sound of zombies howling melodically in a graveyard” (verbatim quote).

**Table 3:** Content of musical hallucinations in 80 participants

Genre	Frequency (%)
Songs from childhood	26 (16.8%)
Classical music	18 (11.6%)
Folk music	19 (12.3%)
Popular songs	26 (16.8%)
Religious music	28 (18.1%)
Songs heard on the radio	5 (3.2%)
Other	16 (10.3%)
Missing	3 (1.9%)
Total	155

### Familiarity

**Fig. 1:**
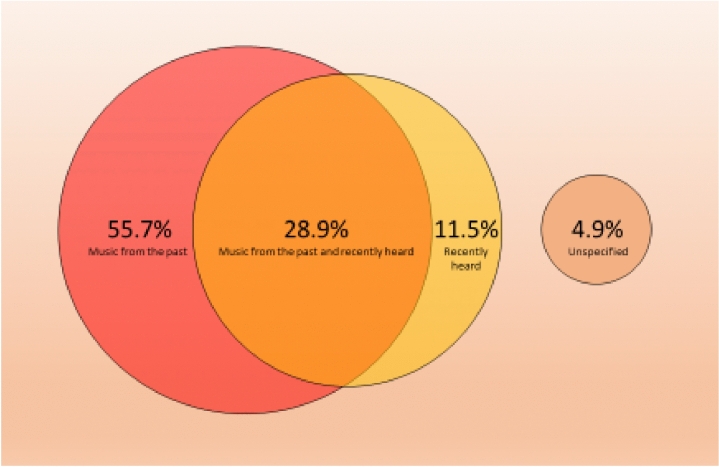
Distribution of types of music perceived (recently heard vs. heard in the past)

Of the 80 participants, 59 (73.8%) reported familiar music, 15 (18.8%) unfamiliar music, and four (5.0%) both. Two participants (2.5%) did not answer the question and were therefore categorised as “missing” (Supplementary material, table S3). In summary, as illustrated in Fig. 1, 61 people (76.3%) reported on (sometimes) hearing familiar music. Of them, 34 (55.7%) reported on hearing only music heard early in life, seven (11.5%) on hearing only music heard recently, and 17 (28.9%) on hearing music from both categories. Three participants (4.9%) did not specify this.

### Musicality

To test the hypothesis whether the brains of musical persons might perhaps be better equipped to produce unfamiliar hallucinated music, we gauged the participants’ self-reported musicality. Of them, 31 (38.8%) considered themselves to be musical, whereas 46 (57.5%) did not. Three people (3.7%) did not answer the question (“missing”). Due to small frequencies, statistical testing was not considered appropriate and results are presented descriptively (Supplementary Material, Table S4).

### Spatial localisation

Of the 80 participants, 45 (56.3%) reported an internal localisation of their musical hallucinations, 22 (27.5%) an external localisation, and eight (10.0%) both, while one (1.3%) was uncertain about this (Table 4). Among those who reported an external localisation, all but two (93.3%) experienced the music with both ears. One of the two remaining participants specified hearing the music at the back of the head, which we interpreted as an extracampine musical hallucination. The other had a hearing problem on the left and reported hearing the music only on the right. Both were specified as “other” (Table 4). Both participants, including the participants who could not recall the spatial localisation and two others with missing data, were excluded from the results (*n* = 4). We conducted two binary logistic regressions to examine whether age and sex predicted the perceived spatial localisation. Given the small number of cases in the group who experienced internal as well as external hallucinations (“both”) (*n* = 8), we did not consider multinominal logistic regression appropriate, and used a binary regression instead. In the first analysis, external hallucinations were compared to internal hallucinations and those perceived in both locations. The overall model was significant (*p* = 0.042), with sex as a significant predictor (*p* = 0.048, OR = 0.275, 95% CI 0.077–0.986), indicating that women were more likely to report external hallucinations than men. In the second analysis, internal hallucinations were compared to external hallucinations and those perceived in both locations, which also showed a significant outcome (*p* = 0.002), with sex again as a significant predictor (*p* = 0.008, OR = 4.944, 95% CI 1.526–16.022), indicating that woman were almost five times more likely to report internal hallucinations than men. In both analyses, age was not a significant predictor. However, the classification table for the first model failed to correctly classify any of the external cases, likely due to the unequal group sizes (external *n* = 21, internal/both *n* = 53). Therefore, these results should be interpreted with caution.

**Table 4:** Spatial localisation of musical hallucinations

Spatial localisation	Number of participants (%)
External	22 (27.5%)
Both ears, both sides	16 (20.0%)
Left ear	3 (3.8%)
Right ear	3 (3.8%)
Internal	45 (56.3%)
External and internal	8 (10.0%)
Other	2 (2.5%)
Unknown	1 (1.3%)
Missing	2 (2.5%)

We did not establish whether localisation changed from onset to the time of the interview, which we observed clinically.

We also conducted two binary logistic regression analyses to examine spatial localisation in relation to the experience of familiar versus unfamiliar music, adjusted for age and sex. The first analysis compared internal hallucinations to external ones and those perceived in both locations. The overall model was significant (*p* = 0.001). Familiar music was not a statistically significant predictor (*p* = 0.062, OR = 3.411, 95% CI 0.939–12.391), although it showed a trend toward significance, suggesting that participants experiencing familiar music were more likely to report an internal spatial localisation. In addition, sex was a significant predictor (*p* = 0.041, OR = 3.582, 95% CI 1.052–12.198), while age was not significant. A second binary logistic regression analysis, comparing external hallucinations to internal hallucinations and hallucinations perceived in both locations, and including familiar versus unfamiliar music as a predictor (adjusted for age and sex), did not show a significant model.

In the group experiencing unfamiliar music, an equal number of participants reported on an internal and external localisation. Among the participants who experienced both familiar and unfamiliar music, none reported an internal localisation (Table 5).

**Table 5:** Spatial localisation compared to familiarity of hallucinated music

	Familiar music	Unfamiliar music	Familiar and unfamiliar music
External	14 (24.6%)	5 (38.5%)	3 (75.0%)
Internal	39 (68.4%)	5 (38.5%)	0 (0.0%)
External + internal	4 (7.0%)	3 (23.1%)	1 (25.0%)

We also compared the spatial localisation of musical hallucinations to clinical diagnoses. Due to the small sample sizes across all groups, we did not consider statistical testing of additive value. Descriptive data are presented in Table 6. Of note, 78.1% of the participants diagnosed with a psychiatric condition reported an internal localisation, compared to 21.9% with an external localization. This may warrant further investigation in larger samples. In contrast, 40.0% of the participants with hearing loss reported an internal localisation, 33.3% an external localisation, and 26.7% both localisations.

**Table 6:** Spatial localisation of musical hallucinations in different clinical diagnoses

	External	Internal	External + internal
Psychiatric	7 (21.9%)	25 (78.1%)	0 (0.0%)
Hearing loss	5 (33.3%)	6 (40.0%)	4 (26.7%)
Structural neurological	1 (33.3%)	1 (33.3%)	1 (33.3%)
Psychiatric + hearing loss	1 (33.3%)	2 (66.7%)	0 (0.0%)
Structural neurological + hearing loss	7 (43.8%)	6 (37.5%)	3 (18.8%)
Psychiatric + structural neurological + hearing loss	1 (33.3%)	2 (66.7%)	0 (0.0%)

Regarding symmetrical versus asymmetrical hearing loss, we found that 66.7% of those with symmetrical hearing loss reported an internal localisation of their hallucinations, 27.8% an external localisation, and 5.6% both types of localisation. In contrast, 33.3% of the participants with asymmetrical hearing loss reported an internal localisation, 25.0% an external localisation, and 41.7% both (See Supplementary Material, Table S5).

### Involvement of other sensory modalities

**Fig. 2:**
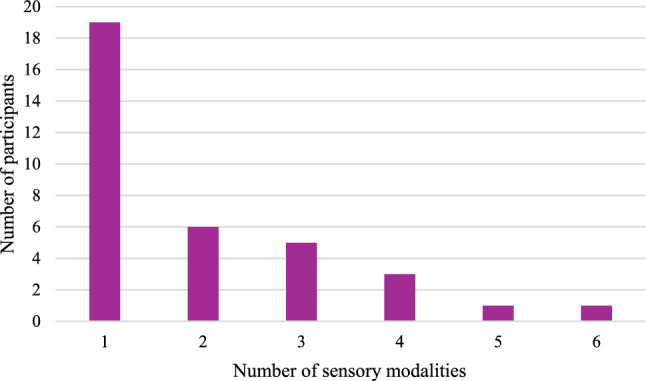
Number of participants who experienced positive disorders of perception in additional sensory modalities, with the x-axis indicating the number of additional sensory modalities. compared to additional hallucinations or sensory distortions experienced in other sensory modalities

We also inquired whether participants experienced hallucinations or sensory distortions in other sensory modalities than the auditory one. In the whole group, 35 people (43.8%) had experienced at least one such phenomenon. To examine whether the presence of experienced hallucinations or sensory distortions in other sensory modalities was associated with the spatial localisation of musical hallucinations, chi-square tests were conducted. For most groups, frequencies were too small to meet the assumptions of the chi-square test, and results are therefore presented descriptively (Table 7). Visual hallucinations were the only type with a sufficient sample size to allow for statistical testing. The chi-square test reached significance (*p* = 0.017), with the majority of participants reporting visual hallucinations having a greater chance to experience an internal spatial localisation (81.5%) than those without visual hallucinations (47.9%). Table 8 shows the frequencies of each reported type of additional hallucination or sensory distortion. Since some people reported on several of these phenomena, the total number of hallucinations and sensory distortions exceeds the number of participants. Of those who reported such additional perceptual phenomena in different sensory modalities, 19 (54.3%) reported one, while 16 (45.7%) reported multiple, with involvement of up to six additional sensory modalities (Fig. 2). As to other types of hallucination experienced *within* the auditory modality, 32 people (40.0%) reported on tinnitus, and 20 (25.0%) on verbal auditory hallucinations. Notably, 13 of the 17 participants with only hearing loss as a clinical diagnosis (76.5%) reported having experienced *no* additional hallucinations or sensory distortions. Of the participants who did report additional hallucinations or sensory distortions, one reported metamorphopsia, one gustatory hallucinations, and one proprioceptive hallucinations.

**Table 7:** Frequencies of hallucinations and sensory distortions in other sensory modalities

Perceptual phenomena	Frequency (%)
Tinnitus	32 (40.0%)
Visual hallucination	27 (33.8%)
Metamorphopsia	7 (5.6%)
Olfactory hallucination	9 (7.2%)
Gustatory hallucination	5 (6.3%)
Tactile hallucination	7 (5.6%)
Somatic hallucination	3 (2.4%)
Sexual hallucination	1 (0.8%)
Thermal hallucination	4 (3.2%)
Kinesthetic hallucination	2 (1.6%)
Proprioceptive hallucination	3 (2.4%)

**Table 8:** Spatial localization compared to reported additional hallucinations

Additional reported hallucinations	External	Internal	External and internal	Total	*p-value*
Visual hallucination	4	22	1	27	0.017*
Metamorphopsia	3	4	0	7	
Olfactory hallucination	1	8	0	9	
Gustatory hallucination	0	4	1	5	
Tactile hallucination	0	7	0	7	
Somatic hallucination	0	3	0	3	
Sexual hallucination	0	1	0	1	
Thermal hallucination	0	4	0	4	
Kinesthetic hallucination	1	1	0	2	
Proprioceptive hallucination	1	2	0	3	

*P* values are based on the *χ*2 test

*P* < 0.05 = *, *P* < 0.01 = **, *P* < 0.001 = ***

Missing values were excluded

Of the 35 participants who reported on additional types of hallucination or sensory distortion, 12 (34.3%) indicated that these occurred simultaneously with the musical hallucinations. This is referred to as a simultaneous multimodal hallucination. [32] Twenty participants (57.1%) indicated that these did not occur simultaneously; they therefore experienced serial multimodal hallucinations. Five participants (14.3%) reported that the timing differed, i.e., sometimes simultaneously and sometimes not. Twelve participants (34.3%) were unsure whether these phenomena coincided in time.

### Burden and control

Of the 80 participants, 52 (65.0%) found their musical hallucinations burdensome, 15 (18.8%) “sometimes burdensome, but not always,” and 10 (12.5%) “agreeable” (with three answers (3.8%) missing). The majority of those who found their hallucinations burdensome reported experiencing them 21–31 days per month (78.8%), as did those who found them sometimes burdensome (80.0%) (Table S6). Of those who reported no additional hallucinations or sensory distortions, 61.2% found their musical hallucinations burdensome. In contrast, among those who found them sometimes burdensome, 78.6% did report additional hallucinations. Notably, the group with both structural neurological changes and hearing loss reported the highest proportion of burdensome hallucinations (93.8%), followed by the hearing-loss group (70.6%) and the psychiatric group (59.4%). The combined psychiatric and hearing-loss group reported hallucinations 21–31 days per month in all cases (100.0%), as did the majority of the hearing-loss (76.5%), structural neurological changes and hearing loss (76.5%), and psychiatric groups (72.7%). (Table S7). Most of those who felt burdened mentioned that they could not stand the noise, felt powerless, had trouble following conversations, had trouble sleeping, and became exhausted, desperate, and depressed. Of them, 67.5%, had no conscious control over their musical hallucinations, in the sense that they could not alter or stop the hallucinations by will, while 20.0% did so to a certain extent, and 12.6% did not answer the question.

Those who reported on having at least some degree of masking or control mentioned varying techniques (Supplementary Material, Table S8). Recorded most frequently––and apparently most effective––were doing something else or talking to someone else (6.3%), as well as concentrating on something else (6.3%). In addition, 34 people (42.5%) tried listening to external sounds from a radio, TV, or other device (including one person who let her spouse hum in her ear). This was successful for 18 of them (22.5% of the total group), in the sense that the musical hallucinations would temporarily stop or recede into the background. Moreover, although it would not alter their hallucinations, 20 people (25%) mentioned that humming or singing along made the music more bearable or simply improved their mood. Of note, none of the participants were able to attain any long-term sustained positive impact on their hallucinations. One special case involved the above-mentioned participant who heard zombies howling in a graveyard. She reported that the noise would sometimes cease when she would slam a door shut. After that it would remain silent for a few moments until she would hear bells tingling in a crescendo-like manner and the howling would return like before.

## Discussion

Our analysis of 80 people who experienced musical hallucinations shows that females were only slightly overrepresented in this group, that––on average––females were significantly older, and that a little over a third of the participants had hearing loss. The music perceived was mostly familiar (71.3%), and chiefly fell into the categories of religious music, children’s songs, and popular songs. Hearing unfamiliar music was not associated with self-reported musicality. Among those participants who experienced familiar music, 55.7% reported hearing only music from the more distant past (e.g. children’s songs), 11.5% music listened to recently, and 28.9% both. Our analysis also showed that the hallucinated music was more often experienced inside the head (56.3%) than outside (27.5%). In the external-localisation group, the music was almost invariably heard binaurally. In addition to their musical hallucinations, 40.0% of the participants experienced tinnitus, 25% voices, and 43.8% at least one type of hallucination or sensory distortion in a different sensory modality. Most of the latter phenomena were visual in nature. As to the number of additional sensory modalities involved, 19 people in this subgroup (54.3%) reported one, and 16 (45.7%) multiple ones (up to a total of six additional sensory modalities). Most of these phenomena combined to form serial multimodal hallucinations (57.1%), and a somewhat smaller proportion (34.3%) simultaneous multimodal (i.e. compound) hallucinations. Moreover, participants with multimodal hallucinations were more likely to experience musical hallucinations inside their head. The same held true for males, who significantly more often than females reported on an internal spatial localisation of the music they perceived. A mediating factor in this may have been the higher rates of hearing loss among females. The participants’ degree of control over their musical hallucinations was generally low, although 20% benefited from this. Especially listening to actual music, redirecting one’s attention, and humming or singing along could interrupt the music or make it more bearable, although these effects were generally only temporary.

### Multimodality

Our finding that a quarter of the participants to our study reported having experienced verbal auditory hallucinations, and almost 50% perceptual disorders in different sensory modalities, is in line with a previous retrospective chart study among 393 people with musical hallucinations [33]. In that study, Golden and Josephs found other types of auditory hallucination in 15.6–50.0% of the cases (in different etiological groups; 47.8% of the participants with a psychiatric etiology), and visual hallucinations in 0–73.9% (21.3% of whom had a psychiatric diagnosis) [33]. And yet the proportion of participants experiencing multimodal hallucinations found by us, as well as the number of sensory modalities involved, is higher than anticipated. An explanation may be that our target population primarily presented to psychiatry (45.0%), as opposed to the group studied by Golden and Josephs [33], although the proportion of psychiatric patients in their sample was still some 40%. Another, more likely, reason may be the systematic way in which we assessed the presence of (other) hallucinations and sensory distortions. Our findings are also in line with the work by Lim et al. [32], who studied a large group of people diagnosed with schizophrenia spectrum disorders and found that unimodal auditory hallucinations (traditionally considered the most common type of hallucination in that group) were outnumbered by multimodal ones. If anything, the high rate of multimodal hallucinations in our sample indicates that the mechanisms underlying musical hallucinations are not always discrete and circumscript. After all, the involvement of multiple sensory modalities hints at the involvement of multiple disparate brain areas within the perceptual network, and/or, alternatively, the involvement of a higher-order brain network that integrates information from these disparate perceptual areas [32].

### Pathophysiology

Although evidence suggests that tinnitus and musical hallucinations share a common neural substrate, musical hallucinations additionally involve the activation of brain regions associated with music and language processing, such as the right-sided Broca’s homolog and the right temporal pole [34]. A systematic review by Jarach et al. reported a tinnitus prevalence of 14% in the general population [35]. Linszen et al. studied 829 people with hearing impairment, of whom 16.2% had experienced auditory hallucinations in the past four weeks, including 5.8% with musical hallucinations. Those with auditory hallucinations had significantly increased percentages of tinnitus in comparison to those without hallucinations (OR 2.0; present in 80.6%) [7]. In our study, 40.0% of participants experienced currently troublesome tinnitus.

The perception, storage, and retrieval of music involves a complex interplay of brain areas that constitute a network that reaches far beyond the superior temporal sulcus and the auditory association cortex [10, 36–38]. An important part of that network is the memory system [22, 39, 40]. Since it is believed that musical hallucinations are often related to learned memories, i.e. perceptual experiences that are stored and accumulated in memory circuits [41], three main, partially overlapping, hypotheses exist on the pathophysiology of these hallucinations. These will be discussed below in the context of our findings.

### Perceptual release

The first hypothesis on the pathophysiology of musical hallucinations is the *perceptual release theory*,[42] which suggests that nonessential data stored in our memory systems can “break through” and “be released into” consciousness due to pathological processes that break down inhibitory mechanisms. Some examples of those processes are structural damage, psychological stress, and substance use/neurotransmitter/receptor changes [43]. When musical and perceptual in nature, the result of such “released” memories is a musical hallucination. Our finding that more than three-quarters of the participants perceived music that was familiar to them (including the 5.0% who heard both familiar and unfamiliar music) may be seen as partial confirmation of the learned-memory hypothesis. Still, 18.8% of these participants claimed that they heard music unfamiliar to them. This begs the question of whether this subgroup perceived music that was truly novel or perhaps heard without consciously registering it (i.e. subconsciously) or previously heard and then forgotten.

### Deafferentiation

A second pathophysiology hypothesis of musical hallucinations is the *deafferentiation theory*, which is a variant of the release theory [44]. It proposes that the release of musical content from memory circuits is caused specifically by sensory deprivation. When normal auditory input diminishes, as in hearing loss (and sometimes also in loneliness), music from memory circuits may then be spontaneously released, and present as a musical hallucination. Because of its analogy to the Charles Bonnet syndrome in visual loss, musical hallucinations attributed to deafferentiation are also referred to as the auditory Charles Bonnet syndrome. In the literature, the deafferentiation* theory* may well be the most cited hypothesis to explain the mediation of musical hallucinations [45]. However, in our study, 50.0% of the participants reported musical hallucinations despite having no documented history of hearing loss. Golden and Josephs had documented hearing loss (not defined) in 105/393 (26.7%) cases [33]. Our higher rates are likely due to our attempts at proactively testing this in as many people as possible. This suggests that, in the remainder of the cases, mechanisms other than deafferentiation play a role, notably those implied by the perceptual release theory, although subclinical hearing loss could conceivably play a role.

### Epilepsy/network dysfunction

A further and third hypothesis is the *ictal theory*. As proposed by Cogan [46], especially episodic, fragmented, and repetitive types of hallucination (such as those experienced by Mr. Van Halen, see Introduction) are considered indicative of ictal attacks. In the case of musical hallucinations, such localised attacks are believed capable of affecting any part of the network involved in the storage and retrieval of music [33]. In a prior study by our group we suggested the existence of four variants of such epilepsy-related musical hallucinations, comprising ictal, interictal, and postictal phenomena, and phenomena elicited artificially with the aid of brain stimulation [47]. Judging by the phenomenological characteristics of the musical hallucinations we analysed (and leaving the brain-stimulation condition out of the equation), the epilepsy-related group was the smallest in our group. We identified only two people who reported content that might be indicative of ictal attacks as described by Cogan [46]. Of note, these two participants were not the same as the three who had a history of epilepsy. Among this total number of five people (6.3%), EEG readings indicated epileptiform activity in only one person (1.3%). Although that does not necessarily refute the hypothesis of an epileptic origin in the remaining four participants, it will be clear that the ictal group was a minority in our study. Incidentally, this finding is in line with a previous review by our group on musical hallucinations and epilepsy [47]. Aberrant network synchronisation and desynchronisation, as in seizures, could however be an underlying shared mechanism. These three hypotheses on the pathophysiology of musical hallucinations are not mutually exclusive. As proposed by Kumar et al. [38], they may be integrated in a model that utilises Bayesian predictive coding to explain how musical hallucinations arise from spontaneous brain activity (or changes in brain activity). This can result from a mismatch between top-down prediction errors (e.g., “what does the brain think it perceives”) and bottom-up error-prediction errors (“which feedback signal does it receive”).

fMRI studies exploring the interaction between epilepsy and musical hallucinations support these network-based neurophysiological models, which are also well-developed for tinnitus [34].

### Spatial localisation

Participants who reported on multimodal hallucinations were more likely to experience their musical hallucinations as inside the head. Of those with an internal spatial localisation, 55.6% were diagnosed with a psychiatric condition. Here, too, the model proposed by Kumar et al.[34] on Bayesian predictive coding may be applicable. In our study, most participants with hearing loss solely experienced musical hallucinations, without involvement of any of the other sensory modalities. Among these participants with hearing loss as the sole identifiable cause, 35.3% reported internal and 29.4% external localisation. In contrast, among those with a psychiatric disorder as the sole cause, 75.8% reported internal and 21.2% external localisation (*p* = 0.418). This finding may support the hypothesis that participants with hearing loss may not have developed the judgment needed to reappraise the perceived source of their hallucinations. On the other hand, multimodal hallucinations in people with first-episode psychosis tend to go hand in hand with greater delusional ideation, and hence with lower levels of insight [48]. This difference may be due to the co-involvement of cognitive networks in schizophrenia spectrum disorders, or the relatively short duration of exposure to hallucinations in younger groups [48]. This topic is in need of further research, as well as the question of whether involvement of the auditory dorsal stream and the planum temporale might play a role in the “projection” of hallucinated music into the outside world. After all, in people experiencing verbal auditory hallucinations in the context of schizophrenia spectrum disorders, this mechanism has been shown to play a crucial role [49]. Another issue to be taken into consideration is that people with musical hallucinations may struggle determining and verbalising whether they actually *experience* the music inside their head or simply have learned that it is *mediated* inside their head. In our study, this was a recurring dilemma for participants.

Tinnitus is also more common and often more severe in individuals with hearing loss, and asymmetric (unilateral) tinnitus is strongly associated with interaural hearing asymmetry, particularly when the difference in hearing thresholds between ears is ≥ 15 dB at two adjacent frequencies. The presence of asymmetric hearing loss increases the likelihood of unilateral or asymmetric tinnitus, but the overall impact and distress from tinnitus are similar between those with unilateral and bilateral tinnitus [50, 51].

### Positively rated musical hallucinations

Since music possesses a unique ability to bring back memories and evoke emotional reactions [22, 52], and also given our fondness of music in general [53], one might expect that hallucinated music in and of itself is often appreciated. Our study does indeed indicate that it is rather the intrusive, disruptive, and uninfluenceable nature of these phenomena that makes it so burdensome. Our finding that 12.5% of the participants rated their musical hallucinations as agreeable is in line with an observation made by the late Oliver Sacks (1933–2015), who saw and received letters from a relatively large number of people with this type of hallucination in his clinical practice, and remarked that “*with the passage of months or years, most people with musical hallucinations find ways of living with them, accommodating to them, so that what was initially felt as so alien and intrusive may become tolerable or even companionable*.”[22] Likewise, those participants who rated their hallucinations as agreeable would often indicate that they had grown accustomed to them, or that things were not as bad as they had been in the past. For example, one of our male participants was glad that he now heard music instead of voices, which in a more distant past had commanded him to jump from an edifice. Another male participant said that the music had become softer over the years and now did not burden him as much as it used to, when it had often prevented him from falling asleep. Likewise, one of our female participants indicated that she found her musical hallucinations “*pleasant and cozy since they did no longer hinder her*,” the reason being that she had been given temazepam to help her sleep. In other words, these three participants rated their musical hallucinations as positive since, by comparison, things had been worse before. However, yet another male participant rated his musical hallucinations as positive in their own right, claiming that he would not want to live without the rock and pop songs that he was continuously being exposed to. His statement is reminiscent of the one made by another patient described by Sacks, who told him that she would miss the music if it would be taken away from her, because “*you see, it is now part of me*.”[22] Since the clinical nature of our study is biased against positive experiences, future analyses of serial measurements in our cohort and other more population based studies may perhaps shed further light on this.

### Symbolic meaning

Musical hallucinations are generally interpreted as “flukes,” as products of spurious brain activity. However, from a psychodynamic point of view one may question the *function* of hallucinated music in people’s lives and probe their meaning to them. The hallucinated music described by Mr. Van Halen in the introduction consisted of repetitive fragments of rock music by the band Led Zeppelin, who were a source of inspiration to Mr. Van Halen in his formative years, and whose work he had studied in great detail [1]. In our study, a case in point is the story of a 76-year-old woman who had started to hear music on the day her husband died (described previously by our group [54]). For over 5 years, she had continuously heard the kinds of hymns, lullabies, pop songs, and classical tunes that the two of them had always been fond of, and instead of singing along with them together, as they had used to, she had now done that by herself. This had given her a deep sense of ongoing connectedness with her late husband, especially after he had appeared to her several times in hypnagogic hallucinations. Eventually the music became so exhausting that she had had to seek help, but it is not hard to see that the type of music as well as its debut had been meaningful to her. Many of the participants in our study likewise experienced music that was meaningful to them, e.g. stemming from childhood or being associated with religious occasions. We did not explore this any further, nor did we explore the moment at which people first started to hear music, although in clinical practice we note this is often at the wake–sleep transition (as has also been noted for tinnitus[55]). These would be interesting topics for future research.

### Limitations

Even though this is the largest prospective cohort study to date on musical hallucinations, a limitation is the still relatively small number of participants. We moreover collected data with the aid of the *MuHa Questionnaire*, which was designed specifically for this purpose, but was not validated and can best be understood as a semi-structured survey. Neither was it independently applied by more than one researcher. Instead, each team member had been trained to administer it. In cases of doubt, issues were discussed with the PIs (J.A.F.C. or J.D.B.). There is a major risk of recall bias with time from onset, with a median of eight years (as noted above, 43.8% recalled onset and clinically we have noticed changes in phenomenology and localisation over time, as described by Dobkin [56].

Another shortcoming of the *MuHa Questionnaire* is that it does not systematically screen for non-musical auditory phenomena other than tinnitus or subdivide instrumental and/or vocal hallucinations, but free text responses did allow for some phenomenological detail. For our present purposes we were able to extract data on verbal auditory hallucinations from the written reports on each participant, but our data on auditory phenomena may therefore well be incomplete. Yet another limitation is that our study was carried out at a psychiatric outpatient clinic. Although we did so in close collaboration with neurologists and otolaryngologists, this may have consequences for generalisability and comparability with other studies. Finally, due to missing data not all analyses were full. In this group of patients, who are often elderly and suffer from psychiatric conditions, it was sometimes difficult to acquire all the necessary information.

## Conclusion

Our analysis of the phenomenological characteristics of musical hallucinations in 80 people yields results that are partly in line with previous studies, while also providing novel insights. In our sample, females were only in a slight majority, and musical hallucinations were more often than not experienced as inside the head (while still fulfilling the formal characteristics of hallucinations). An outside localisation was more common in familiar music and in people with (asymmetric) hearing loss. A novel finding is the large proportion of people who experienced tinnitus and multimodal hallucinations, the latter in up to six sensory modalities. These findings indicate that the underlying pathophysiology of musical hallucinations may often be less discrete and circumscript than has long been suspected. They may also explain why we found a relatively large group of people (28.9%) who had more than one underlying clinical diagnosis. Contentwise, musical hallucinations spanned many genres, with religious music being reported on most frequently. Although 12.5% of the participants rated the hallucinated music as agreeable, this often meant that things were merely better than they had been before. In > 65% of the cases the music was rated as burdensome. Moreover, most of the participants had very little control over their hallucinations, and mostly only fleeting. Future studies should attempt to get deeper phenomenological data close to onset and with frequent follow-up to describe their natural history in more detail.

## Supplementary Information

Below is the link to the electronic supplementary material.

Supplementary file1 (DOCX 22 kb)

Supplementary file2 (DOCX 20 kb)

## Data Availability

All data generated or analysed during this study are included in this article and its supplementary material files. Further data are available at personal request.
