# Supplementary material for: Detailed clinical characteristics of musical hallucinations in 81 patients
Source: J Neurol. 2026 Jul 6;273(8):445. doi: 10.1007/s00415-026-13958-z (PMC13337847; doi:10.1007/s00415-026-13958-z)
Supplement: Supplementary file 1 — Supplementary file1 (DOCX 22 kb) [file 415_2026_13958_MOESM1_ESM.docx]

Natural history, treatment and phenomenology of musical hallucinations

Questionnaire developed by Drs J. A. Coebergh 1,2,3, Prof.Dr. I.E.C. Sommer 4 and Dr. J. D.Blom 5, 6
Version 1.0 Copyright 2010 authors

17 June 2013

REC Nr 13/LO/1586

1 Haga Hospital The Hague, Department of Neurology

2 Ashford/ St.Peter’s Hospital, Chertsey, UK
3 St George’s Hospital, Tooting, UK

4 UMC Utrecht, Division Brains
5 Parnassia Bavo Group The Hague, Clinical Center Adults
6 Universiteit Groningen, Department of Psychiatry

Patient Name:
Date of Birth:
File number:
Name of researcher:
Date of research:
Date of MRI:
Date on which the musical hallucinations began:
Number of years since onset
Measurement number: 1/2/3/4/5


1 Type of music (open question)
1 What musical hallucinations have you heard for the past month? (Please write in detail: interrogate anthem, tearjerkers, carols, hymns, nursery rhymes, classical music, etc.) and is there delay in onset after external stimulation?

Quality of hallucinations
2.1 Does the music sound pure?
     O Yes
     O No
     O Sometimes pure, sometimes false

2.2 Do you hear complete songs?
     O Yes
     O No, only fragments or fragments
     Both O

2.3 is the same music ever repeated?
     O Yes, maximum consecutive times ....
     O No

2.4 Do you hear music you've previously heard?
     O Yes, songs or music that I used to know
     O Yes, songs or songs that I have just heard
     O Other, ......
     O No, I hear .....

2.5 How do you hear it?
     O Through my ears, both sides
     O Through my left ear
     O Through my right ear
     O Inside my head
     O Both inside and outside my head
     O Other, ....
     O Do not know

2.6 From what direction does the music come?
     O From the right side
     O From the left side
     O Straight from the front
     O Right behind
     O Above my head
     O Below
     O From the inside
     O Other, ....
     O Do not know

2. 7 Is the type of music congruent with your mood?
     O Yes, because .....
     O No
     O Other, ....
     O Do not know

3 Frequency
3.1 On how many days of the past month have you experienced musical hallucinations?
1/2/3/4/5/6/7/8/9/10/11/12/13/14/15/16/17/18/19/20/21/22/23/24/25 / 26/27/28/29/30/31

3.2 How many hours per day on average?
1/2/3/4/5/6/7/8/9/10/11/12/13/14/15/16/17/18/19/20/21/22/23/24

 
3.3 What number would you give between 0 (never) and 10 (continuous?)
0/1/2/3/4/5/6/7/8/9/10

4 Control
4.1 Can the musical hallucinations be influenced by distraction?
     O Yes, namely .... The hallucinations are then .....
     O No
     O Do not know

4.2. Do you consciously control the hallucinations?
     O Yes, namely .... The hallucinations are then .....
     O No
     O Do not know

4.3 Do yo influence the choice of music / songs?
     O Yes, namely .... The hallucinations are then .....
     O No
     O Do not know

4.4 Do you ever sing along with musical hallucinations?
     O Yes
     O No
     
4.5 Does it help to sing along with musical hallucinations?
     O Yes, the hallucinations are then .....
     O No

4.6 Do you have influence in other ways on the musical hallucinations?
     O Yes, namely .....
     O No

5 Effects on functioning
5.1 Are your musical hallucinations a nuisance/hindrance/annoyance?
     O Yes
     O No
     O Sometimes, sometimes not

5.2 Do they disturb you at bedtime?
     O Yes
     O No
     O Sometimes, sometimes not

5.3 Do they disturb you in conversation?
     O Yes
     O No
     O Sometimes, sometimes not

5.4 Do they disturb you in concentrating?
     O Yes
     O No
     O Sometimes, sometimes not

5.5 Do they make you feel like you are mentally ill?
     O Yes
     O No
     O Sometimes, sometimes not

5.6 Do they disturb you in in another way?
     O Yes, namely .......
     O No

6 Influence of medication
6.1 What medications are you currently using?
Drug 1: Duration of use: Effect:
Drug 2: Duration of use: Effect:
Drug 3: Duration of use: Effect:
Drug 4: Duration of use: Effect:
Drug 5: Duration of use: Effect:

6.2 Does your medication help against the hallucinations?
     O Yes, Drug (1/2/3/4/5)
     O No
     O Sometimes, sometimes not
     O Do not know

6.3 Which influence has drug (x) on the musical hallucinations?
Repeat the question for any drug which affects the hallucinations!
     O They disappeared
     O They sound less loud
     O They sound further away
     O I hear them less often
     O The quality of the music changes, namely, .......
     O I have more influence on them
     O They bother me less

     O Other, namely .....
     O Do not know

6.4 What medications have you tried in the past against the musical hallucinations? For how long? And to what effect?
Drug 1: Year: Duration of use: Effect:
Drug 2: Year: Duration of use: Effect:
Drug 3: Year: Duration of use: Effect:
Drug 4: Year: Duration of use: Effect:
Drug 5: Year: Duration of use: Effect:

6.5 Have you tried other treatments against the musical hallucinations?
     O Yes, namely .....
     O No

7 Hallucinations last month
7.1 Have the musical hallucinations in the past month changed in severity?
     O Yes, the severity has increased
     O Yes, the severity decreased
     O No, not changed
     O Do not know

7.1.1 Please indicate on a scale of 0 to 10 (0 = no discomfort, 10 = very severe) the severity of hallucinations in the past month has developed? (Indicated by an arrow, for example, from 3 to 5)

0/1/2/3/4/5/6/7/8/9/10

7.2 Have the musical hallucinations in the past month have changed in frequency?
     O Yes, the frequency is increased
     O Yes, the frequency is decreased
     O No, not changed
     O Do not know

7.2.1 Please indicate on a scale of 0 to 10 (0 = never, 10 = always) how the frequency of hallucinations in the past month has developed? (Indicated by an arrow, for example, from 3 to 5)

0/1/2/3/4/5/6/7/8/9/10

7.3 Have the musical hallucinations in the past month changed in volume?
     O Yes, the volume has increased
     O Yes, the volume has decreased
     O No, not changed
     O Do not know

7.3.1 Please indicate on a scale of 0 to 10 (0 = quiet, 10 = deafening) how the volume of hallucinations in the past month has developed? (Indicated by an arrow, for example, from 3 to 5)

0/1/2/3/4/5/6/7/8/9/10

8 Hearing
8.1 Do you suffer from tinnitus?
     O Yes, since the left .....
     O Yes, right since .....
     O Yes, since both sides .....
     O No
     O Sometimes, sometimes not

8.2 Do you suffer from hearing loss?
     O Yes, since the left .....
     O Yes, right since .....
     O Yes, since both sides .....
     O No

8.3 Is the hearing loss stable?
     O Yes
     O No, it worsens
     O No, it improves

8.4 Do you have a hearing aid?
     O Yes, since .....
     O No

8.5 If you have a hearing aid: do you use this?
     O Yes
     O No
     O Sometimes, sometimes not (explanation: ...............................)

8.6 If you have a hearing aid: does this affect the musical hallucinations?
     O Yes, they are less loud
     O Yes, I hear them less frequently
     O Yes, they bother me less
     O Sometimes, sometimes not
     O Other, .......

9 Hallucinations in other modalities
9.1 Have you ever had other types of hallucinations?
     O Images, visions
     O Deformations of normal observed objects
     O Smells
     O Strange taste
     O Unexplained touch on the body
     O Unexplained feelings in the body
     O Sexual hallucinations
     O Strange temperature
     O Feeling as if my body or body parts move
     O Feeling like a body part has another position

9.2 Did these occur simultaneously with with musical hallucinations?
     O Yes, along with musical hallucinations
     O Sometimes simultaneously, sometimes alternating
     O No

9.3 Have you in the past month had other types of hallucinations?
     O Images, visions
     O Deformations of normal observed objects
     O Smells
     O Strange taste
     O Unexplained touch on the body
     O Unexplained feelings in the body
     O Sexual hallucinations
     O Strange temperature
     O Feeling as if my body or body parts move
     O Feeling like a body part has another position

9.4 Did these occur simultaneously with musical hallucinations?
     O Yes, along with musical hallucinations
     O Sometimes simultaneously, sometimes alternating
     O No

10 Musicality
10.1 Are you musical?
     O Yes (explanation: singing, instrument playing, composing, etc.?)
     O No

10.2 Do you suffer from amusia?

     O Yes (explanation: recognition of higher / lower frequencies/ tone / melody / emotional charge; inability to write music, whistle, sing, read, recognize, play music)
     O No

11 Concluding questions
11.1 Are there other health problems that may affect the musical hallucinations?
     O Yes, because .....
     O No

11.2 Social isolation: Do you get help from family, neighbours or home support?

     O Yes (note: .................................................................)
     O No (note: ...............................................................)

11.3 Loneliness: Do you feel lonely or do you feel very lonely?^[[1]](#footnote-1)^

     O Yes (note: .................................................................)
     O No (note: ...............................................................)

11.4 Are there other things that are important for this research?
     O Yes, because .....
     O No

11.5 Do you have any questions?

1. See [Holwerda TJ](http://www.ncbi.nlm.nih.gov/pubmed?term=Holwerda%20TJ%5BAuthor%5D&cauthor=true&cauthor_uid=23232034), [Deeg DJ](http://www.ncbi.nlm.nih.gov/pubmed?term=Deeg%20DJ%5BAuthor%5D&cauthor=true&cauthor_uid=23232034), [Beekman AT](http://www.ncbi.nlm.nih.gov/pubmed?term=Beekman%20AT%5BAuthor%5D&cauthor=true&cauthor_uid=23232034), [van Tilburg TG](http://www.ncbi.nlm.nih.gov/pubmed?term=van%20Tilburg%20TG%5BAuthor%5D&cauthor=true&cauthor_uid=23232034), [Stek ML](http://www.ncbi.nlm.nih.gov/pubmed?term=Stek%20ML%5BAuthor%5D&cauthor=true&cauthor_uid=23232034), [Jonker C](http://www.ncbi.nlm.nih.gov/pubmed?term=Jonker%20C%5BAuthor%5D&cauthor=true&cauthor_uid=23232034), [Schoevers RA](http://www.ncbi.nlm.nih.gov/pubmed?term=Schoevers%20RA%5BAuthor%5D&cauthor=true&cauthor_uid=23232034). Feelings of loneliness, but not social isolation, predict dementia onset: results from the Amsterdam Study of the Elderly (AMSTEL). [J Neurol Neurosurg Psychiatry.](http://www.ncbi.nlm.nih.gov/pubmed/23232034?dopt=Abstract) 2012 Dec 10. [Epub ahead of print] [↑](#footnote-ref-1)
