# Supplementary material for: Detailed clinical characteristics of musical hallucinations in 81 patients
Source: J Neurol. 2026 Jul 6;273(8):445. doi: 10.1007/s00415-026-13958-z (PMC13337847; doi:10.1007/s00415-026-13958-z)
Supplement: Supplementary file 2 — Supplementary file2 (DOCX 20 kb) [file 415_2026_13958_MOESM2_ESM.docx]

**Supplementary material**

1. **MuHa Questionnaire (separate file)**
2. **Supplementary tables S1-S6**

**Table S1 Tone audiometry records for participants with and without hearing aids**

| **Hearing aid** | **Tone audiometry records** | **No tone audiometry records** | ***P-value*** |
| --- | --- | --- | --- |
| Yes | 26 (76.5%) | 8 (23.5%) | *0.002*** |
| No | 23 (62.2%) | 14 (37.8%) | *1.139* |
| **Total** | **52 (65.0%)** | **25 (35.0%)** |  |

P values are based on the χ² test
P < 0.05 = *, P < 0.01 = **, P < 0.001 = ***.
— = Not applicable (one group empty).
Missing values were excluded.

**Table S2 Hearing impairment in males versus females**

|  | **< 35 dB** | **≥ 35 dB** | ***P-value*** |
| --- | --- | --- | --- |
| Male | 13 | 8 | *0.275* |
| Female | 7 | 32 | *<0.001**** |
| Total | 20 | 40 |  |

P values are based on the χ² test
P < 0.05 = *, P < 0.01 = **, P < 0.001 = ***.
— = Not applicable (one group empty).
Missing values were excluded.

**Table S3 Clinical diagnoses versus familiarity with the music perceived**

|  | **Familiar**  **music** | | **Unfamiliar music** | **Familiar and unfamiliar music** | **Total** | ***P-value*** |
| --- | --- | --- | --- | --- | --- | --- |
| Psychiatric disorder | 26 (78.8%) | 6 (18.2%) | | 0 (0.0%) | 33 (100.0%) | *<0.001**** |
| Hearing loss | 11 (64.7%) | 5 (29.4%) | | 1 (5.9%) | 17 (100.0%) | *0.011** |
| Structural neurological changes | 3 (100.0%) | 0 (0.0%) | | 0 (0.0%) | 3 (100.0%) | — |
| Psychiatric disorder + hearing loss | 3 (100.0%) | 0 (0.0%) | | 0 (0.0%) | 3 (100.0%) | — |
| Structural neurological changes + hearing loss | 10 (58.8%) | 4 (23.5%) | | 3 (17.6%) | 17 (100.0%) | *0.080* |
| All three causes | 3 (100.0%) | 0 (0.0%) | | 0 (0.0%) | 3 (100.0%) | — |
| Other | 0 (0.0%) | 0 (0.0%) | | 0 (0.0%) | 1 (100.0%) | — |
| No identifiable cause | 3 (100.0%) | 0 (0.0%) | | 0 (0.0%) | 3 (100.0%) | — |

P values are based on the χ² test
P < 0.05 = *, P < 0.01 = **, P < 0.001 = ***.
— = Not applicable (one group empty).
Missing values were excluded.

**Table S4 Reported familiarity of the music versus musicality of the participants**

| **Musicality** | **Familiar music** | **Unfamiliar music** | **Familiar and unfamiliar music** | ***P-value*** |
| --- | --- | --- | --- | --- |
| Musical | 25 (42.4%) | 5 (33.3%) | 1 (25.0%) | *<0.001**** |
| Non-musical | 33 (55.9%) | 10 (66.7%) | 2 (50.0%) | *<0.001**** |

P values are based on the χ² test
P < 0.05 = *, P < 0.01 = **, P < 0.001 = ***.
— = Not applicable (one group empty).
Missing values were excluded.

**Table S5 Spatial localisation of musical hallucinations in symmetrical versus asymmetrical hearing loss**

| **Spatial localisation** | **Symmetrical hearing loss** | **Asymmetrical hearing loss** | ***P-value*** |
| --- | --- | --- | --- |
| External | 10 (25.0%) | 3 (25.0%) | *0.052* |
| Internal | 24 (60.0%) | 4 (33.3%) | *<0.001**** |
| External and internal | 2 (5.0%) | 5 (41.7%) | *0.257* |
| Other | 2 (5.0%) | 0 (0.0%) | — |
| Unknown | 1 (2.5%) | 0 (0.0%) | — |
| Missing | 1 (2.5%) | 0 (0.0%) | — |

P values are based on the χ² test
P < 0.05 = *, P < 0.01 = **, P < 0.001 = ***.
— = Not applicable (one group empty).
Missing values were excluded.

**Table S6 Techniques mentioned by participants to exert control over their musical hallucinations**

| **Technique** | **Frequency (%)** |
| --- | --- |
| Listening to external music/sounds  Talking to someone, doing something | 34 (42.5%)  5 (6.3%) |
| Thinking of something else | 5 (6.3%) |
| Substance use | 2 (2.5%) |
| Avoiding actual music | 1 (1.3%) |
| Realising that the music stems from within | 1 (1.3%) |
| Praying | 1 (1.3%) |
| Mindfulness | 1 (1.3%) |
| Breathing harder | 1 (1.3%) |
